# Supplementary material for: The Depression, Anxiety, Stress Scales-21: Principal component analysis and evaluation of abbreviated versions in young adults with temporomandibular disorders
Source: PLoS One. 2025 Jan 27;20(1):e0316703. doi: 10.1371/journal.pone.0316703 (PMC11771923; doi:10.1371/journal.pone.0316703)
Supplement: S1 Appendix — (DOCX) [file pone.0316703.s001.docx]

**S1 Appendix**

As mentioned in the CFA procedure, when the bifactor model was applied for those factors with two variables, identification problems would occur. Several solutions have been proposed to address the issue.

When evaluating a model, two crucial things to consider are the information in the data and the required parameters for estimation. The heart question of identification problems was whether the information data provided could estimate the parameter uniquely. For example, when three indicator variables were in one factor, the covariance matrix (3*3) showed six pieces of information and the number of parameters required to be estimated in this model was also six. Thus, the information was just enough to estimate the parameters. This model was called the just-identified model. If there were four indicator variables, the covariance matrix (4*4) presented ten pieces of information, and there were only eight parameters to be estimated. The information data provided exceeded the number of parameters to be estimated, making it over-identified. Similarly, when there were only two variables in one factor, the covariance matrix (2*2) showed three pieces of information, but there were four parameters to be estimated. The identification problem arose due to insufficient information for uniquely estimating the parameters in this model.

Factor with two variables could be assessed when the correlation was strong between factors because a structural relation can be defined as Factor A = a * Factor B and one parameter has been represented in this equation. Therefore only three parameters were needed for estimation in this case. Two variables could provide three pieces of information, making it just-identified. However, the bifactor model does not allow the factors to be correlated. The identification problem could not be solved in this way.

Solutions for solving the above identification problem in the bifactor model were proposed. Beaujean (2014) suggested setting the two variable’s loadings equal to each other if the error variances of these two variables were not correlated. Another solution recommended in the AMOS IBM guideline was to set the error variance equal to 0.05, making the factor loading equal to 0.95. Both methods reduced the number of parameters needed to be estimated and made the model just-identified. However, these methods may still have problems in estimating the standard error and p-value, making the results unreliable.
