# Supplementary material for: The Depression, Anxiety, Stress Scales-21: Principal component analysis and evaluation of abbreviated versions in young adults with temporomandibular disorders
Source: PLoS One. 2025 Jan 27;20(1):e0316703. doi: 10.1371/journal.pone.0316703 (PMC11771923; doi:10.1371/journal.pone.0316703)
Supplement: Attachment — Submitted filename: Response to reviewers submitted.docx [file pone.0316703.s003.docx]

**Response to reviewers**

We wish to express our great appreciation to the Editors and Reviewers. Here’s our responses.

**ACADEMIC EDITOR:**

1. Please follow the recommendations of the reviewers. In addition, please include recommendations for further validation of this simplified instrument, based on criterion validity and optimisation of the instrument (sensitivity or specificity).

**Response:**

Criterion validity (correlation between the abbreviated version and the DASS-21) has been evaluated using concurrent validity. We have revised the manuscript by emphasizing it. For optimisation of the instrument (sensitivity or specificity), we have added the recommendation for such validation of the sensitivity and specificity by comparing the Korean DASS-12 version with the Diagnostic and Statistical Manual of Mental Disorders [48], which remains the “gold standard” for diagnosing mental disorders. We appreciate the suggestion.

2. Furthermore, in the flowcharts, please specify what the values or coefficients placed on the arrows of the structural diagrams correspond to. This can be done at the bottom of the figure.

**Response:**

A note has been added at the bottom of the figure.

3. Include in the discussion the advantages and disadvantages of applying principal component analysis vs. parallel analysis, or whether it is advantageous to include both.

**Response:**

Actually, parallel analysis (PA) [31] is one method to determine the number of factors or components to retain in the initial factor analysis. It was first mentioned in the methods section, “A combination of the parallel analysis (PA) method, minimum average partial (MAP) method [32], and scree plot was used to identify the number of components to be retained [33]”. There are many ways to determine the number of factors or components, and as the latest evidence suggest, a combination of these methods was used to generate a more reliable result.

In the discussion section, the reason why principal component analysis (PCA) was chosen and the difference between PCA and exploratory factor analysis (EFA) was discussed, illustrating the usage of these two methods. In our study, we used PA as one of the methods to determine the appropriate number of principal components to be extracted during PCA progress. They should not be compared.

Thank you for your valuable suggestion.

**Reviewer #1**:

1. Citations and references such as “Lee et al., 2019” should not be included in the manuscript abstract, specifically in 14 spacing. It is suggested that any external references be removed to ensure that the abstract is self-sufficient and fully understandable without recourse to the full text.

**Response:**

Deleted. We appreciate your suggestion.

2. In the discussion, paragraph 8, line spacing 6, there has been an error in the automatic referencing of the manuscript, reflected in the message “Error! Reference source not found”. It is necessary to review the referencing system and verify that all citations are properly linked to their respective sources in the bibliography or reference list.

**Response:**

Reference 46 has been added. Thank you for picking this up.

3. Check bibliographic references 1,3,4,5,6,7,8,9,10,12,13,14,15,16,17,19,20,21,22,24,25,26,29-43 y 45

Please note the attached Example taken from instructions to the author from Plos One journal: Devaraju P, Gulati R, Antony PT, Mithun CB, Negi VS. Susceptibility to SLE in South Indian Tamils may be influenced by genetic selection pressure on TLR2 and TLR9 genes. Mol Immunol. 2014 Nov 22. pii: S0161-5890(14)00313-7. doi: 10.1016/j.molimm.2014.11.005.

Note: When providing a DOI, follow the format of the example above with the label and full DOI included at the end of the reference (doi: 10.1016/j.molimm.2014.11.005). Do not provide an abbreviated DOI or URL.

**Response:**

We have revised the reference format according to the requirements. Thanks for your reminder.

**Reviewer #2**:

1. In relation to the document read, I consider the great effort made by the authors in proposing this paper could improve the following: Discussion: The discussion could be more exploited by comparing with other studies done in this regard.

**Response:**

We have added one paragraph discussing and comparing the relevant studies.
